# The Ideal Reporter

**Published:** 1901-05-15

**Authors:** C. M. Wright

**Affiliations:** Cincinnati, Ohio


THE
### DENTAL REGISTER.

Vol. LV. May 15, 1901. No. 5.

### THE IDEAL REPORTER.

BY C. M. WRIGHT, D.D.S., CINCINNATI, OHIO.

I have been pondering upon the qualities of an ideal reporter; not only of dental and medical societies, court proceedings and great speeches, but of after dinner remarks, social functions, interviews and all the other occasions where an account is to be printed in a newspaper or scientific journal for the benefit of readers, or for the preservation of valuable ideas, hints or suggestions.

There are a great many men and women who have acquired the art of short-hand writing and who are called stenographers. A stenographic representation of proceedings is supposed to be an accurate statement of all that has been said at a certain time. It is supposed to be as complete in its way as a photographic picture, and perhaps it is. The photographs of a trotting or running horse are pictures of the moving animal so instantaneously caught that they do not give impressions of what we think we see in the real running horse, and yet we dare not criticise the photographs, because they are supposed to be very official impressions taken by a well constructed camera, and not by the camera-like organ of sight with its optic nerve attachments and connections with the perceptive faculty of the brain. An artist, whether he be a disciple of the realist or of the impressionist school of painting, could hardly use the photographic pictures as models for his moving horses. We demand of him representations of what we think we see—a presentation of our ideas of running horses.

Can I use this as an illustration of what the reporter should give us?

The ideal reporter, then, should not be a short-hand writer, or a stenographer, or any other machine. He should be an accomplished observer with cultivated powers of attention, and artist enough to be able to make pen pictures of the remarks, speeches, sayings or what not of the actors at the meeting which he proposes to "report." While reading this "report" we should be mentally transported to the meeting; we should sniff the air, hear the tones, see the speaker and his audience and get the "gist" of the subject, with a word or a sentence characteristic of the speaker. The ideas of this and that member and the occasion would be before us in a harmonious, readable and picturesque article.

How few stenographic reports of dental meetings approach this expectation!

The plan generally, is to employ a stenographer—nothing will do but a stenographer, and this machine must take down in unreadable dots, lines and curves every vibration that taps upon his tympanum. Then the "transcribing" follows, and if all the lines and dots and curves can be made out we have a "report." This passes into the hands of an editor, who, even if present at the meeting, has not depended upon his own impression of what he has heard or seen, but upon the photographic, stenographic transcript, and he tears his hair in a fruitless endeavor to make readable matter out of the conglomerate mass of words.

Is it any wonder that we have garbled statements, false impressions, twaddle and nonsense from some of the best thinkers in our profession in some of our printed "discussions"?

I learned recently that the highest priced reporters of great metropolitan newspapers were long-hand writers, and not stenographers. Such an one is an artist, and his pictures live and are true. He paints, and the reader gets a correct idea of what was said and done. He has no transcript or editor.

In our profession there is a need of just such work. A vast amount of valuable suggestion is lost by our machine methods. Some debaters write their remarks afterward, to avoid garbled statements; others insist upon correcting the "proofs" of their speeches before allowing them to be published; still others, in disgust for past indignities, decline to place themselves at the mercy of stenographers, and only rise to their feet when assured that there is no "chile takin' notes" present.

I am not misstating conditions. There is room for improvement, and I may take the liberty, I hope, of suggesting a plan: Let the younger men of the profession, whenever they attend dental meetings, come prepared to take long-hand notes. The exercise itself is a valuable one in the practice of writing and in the cultivation of attention, perception and judgment. In a little while we should have ample material from which to select accomplished literary reporters. Avoid stenography and all machine methods, for, as it seems to me, the very dependence on short-hand interferes with the free use of the higher faculties of the mind, so necessary to this art.

I am not making careless or random statements, but offering the results of reflection and experience—not pyrotechnics this time, but a steady torch, lighted at the altar of reason.

